# Medical error in neonatal units: an Italian survey on national frequency and types of error

**DOI:** 10.1186/s13052-026-02306-6

**Published:** 2026-08-11

**Authors:** Luca Massenzi, Roberta Parladori, Luigi Gagliardi, Massimiliano Ciantelli, Gloria Brigiari, Francesco Crispino, Camilla Gizz, Nunzia Decembrino, Elena Sorrentino, Genny Raffaeli, Sabrina Palamides, Rocco Agostino

**Affiliations:** 1 Neonatology and NICU, Ospedale Provinciale, Bozen, Italy; 2 NICU, IRCCS S. Orsola, Bologna, Italy; 3 Neonatology and Pediatric, Ospedale Versilia, Viareggio, Italy; 4 Neonatology, Azienda Ospedaliera Universitaria, Pisa, Italy; 5 Unit of Biostatistics, Epidemiology and Public Health, Department of Cardiac, Thoracic, Vascular Sciences and Public Health, University of Padua, Padua, Italy; 6 NICU and Pediatry, ASReM, Ospedale A. Cardarelli, Campobasso, Italy; 7 Neonatology and NICU, Ospedale S. Eugenio, Rome, Italy; 8 NICU, Policlinico G. Rodolico S. Marco, Catania, Italy; 9 Neonatology and NICU, Ospedale S. Pietro Fatebenefratelli, Rome, Italy; 10 NICU, Fondazione IRCCS Ca’ Granda Ospedale Maggiore Policlinico, Milan, Italy; 11 Department of Clinical Sciences and Community Health, Università degli Studi di Milano, Milan, Italy; 12 Neonatology and NICU, Ospedale S. Giovanni Addolorata, Rome, Italy; 13 Ethics Committee for drugs, IRCCS Ospedale Pediatrico Bambino Gesù, Rome, Italy

**Keywords:** Medication errors, Neonatal Units, Drug safety, Risk management, Neonatal pharmacology, Patient safety, Error reporting

## Abstract

**Background:**

Neonatal Units (NUs) are high-risk environments for medication errors (MEs), mainly due to complex weight/gestational age-based dosing and frequent off-label drug use. Although the incidence of serious harm is low, medication errors remain a major safety concern. The aim of this study was to have a snapshot of MEs errors within Italian NUs.

**Methods:**

The Neonatal Pharmacotherapy Study Group and the Risk Management and Simulation Study Group of the Italian Society of Neonatology (SIN) conducted a national cross-sectional web-based survey between 2022 and 2023. Responding NUs were stratified into two groups: those reporting fewer than five MEs per year (< 5 MEs/y )and those with five or more (≥ 5 MEs/y). Descriptive and inferential statistics were applied.

**Results:**

Prescription (33%) and drug preparation (29%) were the most frequently reported error types. The use of digital prescribing tools was higher in centers reporting fewer errors. Double-checking procedures limited to high-alert drugs was associated with higher error rates. 80% of NUs did not have a structured annual error report, and 55% failed to re-evaluate or implement corrective actions after identifying errors. Only 21% of NUs systematically informed families of therapeutic errors; as reasons for non-disclosure many centers citing the absence of harm or fear of legal/professional consequences.

**Conclusions:**

While some centers adopt proactive safety strategies, others use incomplete protocols or outdated tools. The lack of systematic error reporting underscores the need for a cultural shift toward a constant analysis of this problem and a more continuous surveillance and evaluation of implemented safety strategies. A unified, anonymous digital reporting system could support error monitoring and targeted interventions at the local and national levels.

**Supplementary Information:**

The online version contains supplementary material available at https://doi.org/10.1186/s13052-026-02306-6.

## Introduction

Therapeutic errors pose a significant challenge in the paediatric setting, particularly in neonatal units (NUs), where newborns are often critically ill and require several medications. The neonatal population is particularly vulnerable to such errors due to the complexity of weight-based dosing, the need for precise calculations, and the frequent use of off-label medications [1].

Medication errors (MEs) that could result in significant harm are three times more likely to occur in NUs compared to adult wards [2]. Error rates range from 4 to 35 per 1000 patient-days and from 5.5 to 77.9 per 100 medication orders in different reports. The rate of preventable adverse drug events in NUs is 0.86 per 1000 doses, with anti-infective agents being the most implicated drug class [3].

Pharmacological therapy involves multiple professionals, including neonatologists, pharmacists, and nurses. MEs can occur at various stages of the therapeutic process, including prescription, distribution, preparation, and administration. Prescription and administration errors represent the most common phases subjected to errors, with dosing being the most frequent type [3].

Error reporting is not uniformly performed among Italian NUs, and updated data are lacking. The aim of this study was to describe the frequency and the types of pharmacological errors among Italian NUs belonging to the Italian Society of Neonatology (SIN). Additionally, it seeks to analyse the underlying causes and associated risk factors to evaluate the relevance of the safety practices implemented across participating centers.

## Methods

### Study design

The Neonatal Pharmacotherapy Study Group and the Risk Management and Simulation Study Group of SIN conducted a national cross-sectional survey distributed among all the 117 NUs of SIN from January 2022 to May 2023. Participation was voluntary and anonymous, informed consent was implied upon acceptance and completion of the survey. A reminder was sent to non-responders every 2 weeks for a maximum of three times; if no answer was received, participants were contacted by phone.

### Questionnaire

The survey consisted of a 44-item web-based questionnaire investigating two main domains: therapeutic and clinical errors. All questions were loaded on the Google Form Website (Google Inc., CA/USA), a free tool for creating online survey forms. This paper focuses exclusively on the treatment-related section (Questions 1–27). The full questionnaire is available as supplementary material (Appendix 1).

The items addressed several key areas, including drug prescription practices, dose calculation methods - using resources such as the Neofarm-SIN 2020 mobile app (IdeaCongress srl, Rome, Italy) [4] and *2009 SIN-Manual of Neonatal Pharmacotherapy *[5] - medication preparation and administration, double-check procedures, adopted protocols and digital technologies, error reporting systems, and communication to the families.

In the survey, drugs were categorized as High Alert (HA) drugs, defined as drugs that can have a higher risk of causing injury or harm (e.g., heparin, insulin), Look Alike Sound Alike (LASA) drugs, defined as drugs that can be confused for similarities in their name or packaging, and Emergency drugs (e.g., adrenaline), (Appendix 2).

### Data management and analysis

Responses were collected and processed anonymously. Data from each center were evaluated together to calculate overall percentages. Subsequently, the centers were stratified into two groups based on their self-reported frequency of MEs in 2020:

- **Group 1**: centers reporting fewer than 5 errors per year (< 5 MEs/y).
- **Group 2**: centers reporting 5 or more errors per year (≥ 5 MEs/y).

This cutoff was chosen pragmatically because the survey collected error frequency as an ordinal categorical variable (< 5, 5–10, 10–20, > 20 errors/year), and the number of centers in the higher categories was limited. Dichotomization at 5 MEs/year therefore allowed a more robust comparison between centers reporting very low error frequency and those reporting at least 5 MEs/year.

NUs were also categorized by geographic area into North, Center, South and Islands.

### Statistical analysis

Descriptive statistics were used to summarize the characteristics of the participating NUs and their responses. Categorical variables were reported as absolute frequencies and percentages. Comparative analyses were conducted between Group 1 and Group 2 to evaluate differences in therapeutic practices, potential correlations with error frequency or events reporting systems, and risk management strategies.

Associations between center characteristics (e.g., geographical distribution, number of NU beds) and error frequency were analyzed using univariable logistic regression models to identify factors independently associated with higher error rates. Odds ratios (ORs) with 95% confidence intervals (CIs) were reported. A p-value < 0.05 was considered statistically significant. All analyses were performed in R, version 4.3.3.

## Results

Eighty-nine of 117 contacted centers responded to the survey, resulting in a response rate of 76%.

Forty-eight NUs (54%) were allocated in Group 1, and 41 NUs (46%) in Group 2 according to self-reported frequency of MEs. Based on geographic area, NUs were divided into North (35), Centre (17), South and Islands (37).

### Geography

The national distribution of reported errors shows that the South and Islands declared the highest rate of errors (42%), followed by the North (39%) and the Centre (19%). Focusing on error frequency, South and Islands declared mainly <5e/y (62%), while among centers with ≥5e/y, North showed the highest values (66%) (Table 1).

NUs located in the northern regions of Italy have markedly higher odds of reporting ≥ 5 therapeutic errors per year compared to those in the South and Islands OR14.5, 95% CI4.89-48.6, *p* < 0.001. (Table 2).

**Table 1:** Distribution of centers based on error frequency (< 5 vs. ≥ 5 errors/year) and geographical area

Geographical Area	Total Centers	*Centers <5e/y (n = 48)*	Percentage < 5 e/y	*Centers ≥5e/y (n = 41)*	Percentage ≥ 5e/y
North	35	8	17%	27	66%
Center	17	10	21%	7	17%
South - Islands	37	30	62%	7	17%
Total	**89**	**48**	**100%**	**41**	**100%**

**Table 2:** Association between geographic area and risk of ≥ 5 errors/year (univariable logistic regression, n°89)

Geographic area	OR	95% CI	*p*-value
South & Islands (Ref.)	–	–	–
Centre	3.00	0.84–11.0	0.09
North	14.5	4.89–48.6	**< 0.001**

Ref. = reference category; OR = odds ratio; CI = confidence interval. Statistically significant values in bold

### Medication chart

More than two-thirds of centers (66%) reported using a paper-based medication chart (shared between physicians and nurses), with a higher prevalence of 71% in centers with a lower error rate compared to those in Group 2 (61%). Daily medications were computer-based in 30% of all centers, and the averages did not differ between the two error frequency groups. A medication chart was not present in 2.2% of cases.

### Prescription-dose calculation

Most NUs (72%) declared using the *2020 App Neofarm-SIN *[4] mobile version for drug prescription, with a higher percentage among centers reporting fewer errors (79% in Group 1 vs. 63% in Group 2). Approximately 20% of centers used the *2009 Italian Manual of Neonatal Pharmacotherapy *[5], more frequently in Group 2 NUs (24%) compared to those in Group 1 (15%).

In 75% of units, physicians calculated drug doses manually or using a calculator; 21% reported using dedicated dosing software.

### Double check – drug prescription

Prescription verification by a second physician was performed only for HA drugs in 43% of NUs, with no significant difference in error frequency between the two groups. Only 20% of NUs applied a double-check for all prescribed medications, more commonly in Group 1 (25%) than in Group 2 (15%). Notably, 36% of centers reported not performing any prescription verification (35% in Group 1 and 37% in Group 2). (Table 3)

**Table 3:** Double-check procedures during drug prescription

Double-check procedure	Overall *n*°89	< 5 errors *n*°48	≥ 5 errors *n*°41
No check	32 (36%)	17 (35%)	15 (37%)
All drugs checked by second MD	18 (20%)	12 (25%)	6 (15%)
HA drugs checked by second MD	38 (43%)	19 (40%)	19 (46%)
HA drugs checked by Pharmacist	1 (1%)	0 (0%)	1 (2.4%)

Data are presented as n (%). MD = Medical Doctor - HA = high-alert drugs

### Double check - drug preparation

A second nurse made a double check for HA (38%) drugs or for all medicines (34%) preparation. Differences between groups were marked: in Group 2, verification was more often limited to HA drugs (59%) and three times less to all drug preparations (20%). In contrast, Group 1 performed double check preparation for all drugs in 46% of cases: more than twice the rate seen for HA drugs alone (21%). NUs that limited preparation double checks to HA medications had significantly higher odds of reporting frequent MEs than NUs that applied double checks to all medications (OR 6.00; 95% CI 2.11–18.6 *p* = 0.001). (Tables 4 and 5)

**Table 4:** Double-check procedures during drug preparation

Double-check procedure	Overall (*n*°89)	Group 1 (*n*°48)	Group 2 (*n*°41)
No check	24 (27%)	15 (31.3%)	9 (22%)
All drugs checked by second Nurse	30 (33%)	22 (45.8%)	8 (19.5%)
HA drugs checked by second Nurse	34 (38.2%)	10 (20.8%)	24 (58.5%)
HA drugs checked by Pharmacist	1 (1.1%)	1 (2.1%)	0

Data are presented as n (%). HA = high-alert drugs

**Table 5:** Association: preparation double-check and ≥ 5 errors/year (univariable logistic regression, n°89)

Double-check procedure	OR	95% CI	*p*-value
All drugs (Ref.)	–	–	–
No check	1.65	0.52–5.36	0.40
Only HA drugs	6.00	2.11–18.6	**0.001**

Ref. = reference category; OR = odds ratio; CI = confidence interval. Statistically significant values in bold

### Protocols for preparation

Protocols for drug preparation and administration were identified for HA drugs in 43% of NUs, for emergency drugs in 18% and for LASA drugs in 4.5%, respectively. Among Group 2, 41% declared standardized procedures for HA drugs, 24% for emergency drugs, and 2.4% for all medications. 16% of NUs reported the absence of any preparation protocols.

### Drug dilution

Guidelines for drug dilution were available in 65% of NUs, with slightly higher adoption in Group 2 (71%) compared to those in Group 1 (60%).

In cases requiring patient-specific dilution modifications, changes were made jointly by the physician and nurse in approximately 90% of centers and by the doctor alone in 11% of NUs.

### Drug preparation and administration

Over 90% of NUs had professional nurses preparing drugs; 3.4% of centers reported having dedicated nurses assigned exclusively to this task. Only one NU used a robotic preparation system.

For the Parenteral Nutrition (PN) bag, it was often prepared by the hospital pharmacy (in more than 50% of centers); however, in 30% of NUs, it was a specific nurse’s competence, and in 4,5% of cases, there were dedicated nurses.

In 79% of NUs, those who prepared the medication were also responsible for its administration, with no significant differences in error frequency between the two groups.

### Identification system

In most of NUs (66%), medications were prepared and administered immediately without labeling: this practice was reported by 73% of Group 1 and 59% of Group 2. Approximately 26% of NUs implemented various forms of identification, such as associating drug prescriptions with the patient’s identification bracelet. In contrast, a smaller percentage, specifically 5.6%, employed bedside barcode systems to identify single-dose medications administered to patients.

## Errors report

### Errors reporting practices

In 80% of NUs, therapeutic error notification relied on voluntary incident reporting systems, with a higher percentage among Group 2 (85%) than those with fewer errors (73%). Units allowing anonymous error reporting were more likely to be in the high-error category (OR 3.11; 95%CI 1.32–7.58, *p* = 0.011).Additionally, 16% of centers used retrospective review of medical records as a method to increase error detection and consequently error reporting. The reporting format included digital tools in 39.3% of centers, paper forms in 21.3%, and email in 7.9% of centers.

### Frequency and type of errors

Among respondents, 54% reported fewer than five errors per year, 35% declared between five and ten errors, 9% reported ten to twenty, and approximately 2% indicated more than twenty errors during the year of analysis.

The most frequently reported types of errors involved the prescription process (33%), followed by drug preparation (29%), transcription (20%), and administration (17%).

**Fig. 1:**
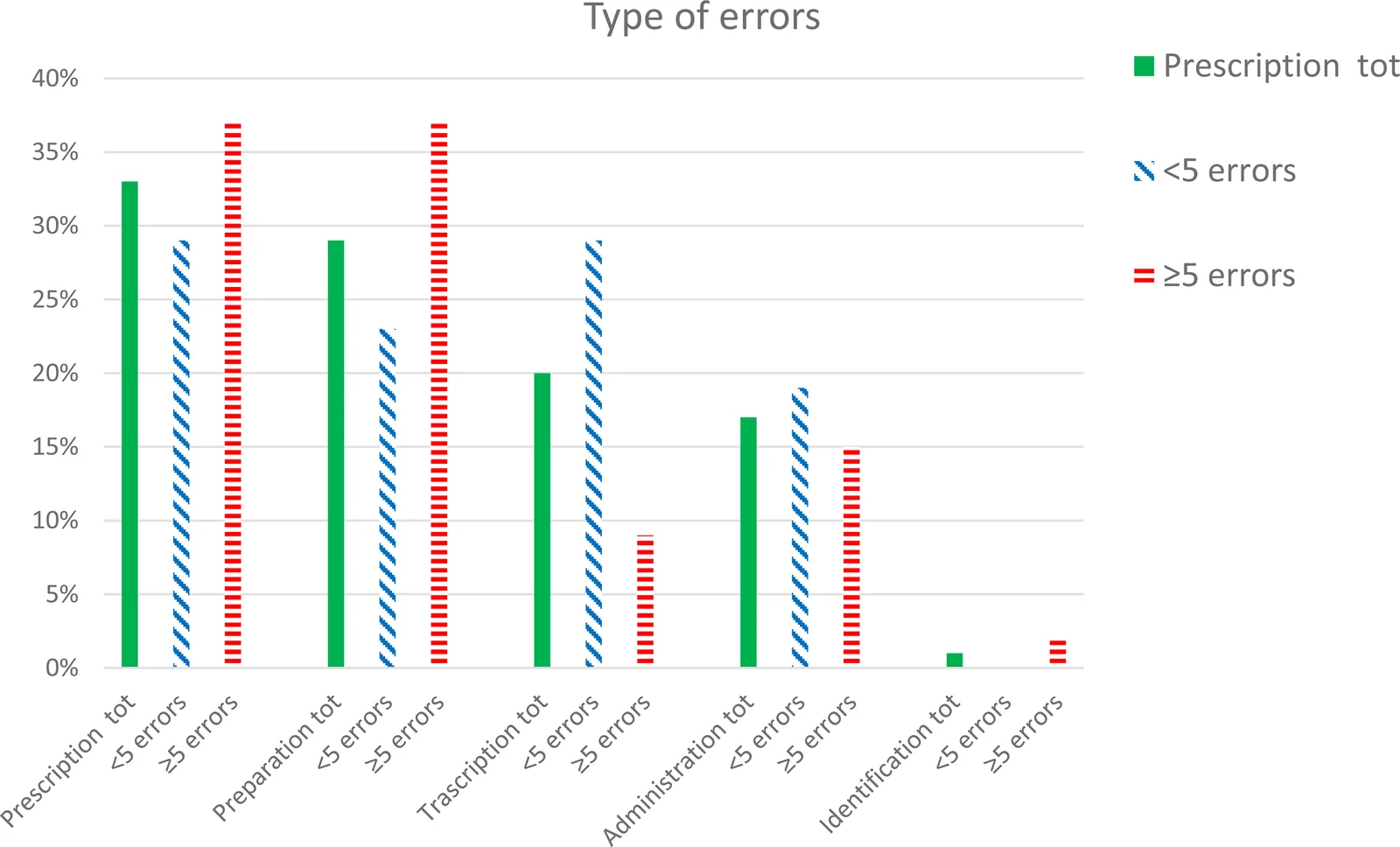
Types of errors: total percentage, percentage in < 5 errors/year Units and ≥ 5 errors/year Units

When stratifying by error frequency group, Group 1 most commonly reported prescription and transcription errors (29% each), followed by preparation (23%) and administration errors (19%). In contrast, Group 2 reported that prescription and preparation errors were equally represented (37%), followed by administration (15%) and transcription (9.8%), (Fig. 1). The night shifts, in comparison with day shifts, showed an increased risk in developing 5 or more errors (OR1,5;95%CI 0,23 − 10,4, *p* = 0.7).

### Communication of errors to families

Around 20% of NUs consistently informed families when therapeutic errors occurred. In 35% of centers, disclosure occurred only if the error had clinical implications requiring assistance or therapeutic adjustments. An additional 27% of NUs contacted families only when there were consequences for the patient. Among respondents, 17% reported rarely communicating errors, 9% said they never or seldom did so, and 7.9% indicated that they disclosed errors only occasionally.

Among centers that did not routinely communicate therapeutic errors to parents, 42% reported the absence of clinical consequences as the most cited reason. In 33.7% of cases, the decision not to disclose was motivated by concern over professional repercussions. Concerns regarding potential legal liability were reported by 15.7% of NUs, while 9% reported hesitance to undertake additional burdens or responsibilities due to the disclosure process.

### Monitoring and intervention

NUs that declared the use of an electronic system for therapeutic error reporting were significantly more likely to be classified in the high-error reporting group compared to those without such systems (OR 4.75; 95% CI1.94–12.3, *p* < 0.001).An annual error report was not a standard procedure in 81% of centers, and 55% of centers did not re-evaluate any corrective actions applied after an error occurred.

## Discussion

Pharmacological treatment in NU requires precise doses and multiple dilutions [6]. Fortunately, the rate of MEs resulting in serious harm is low, and the percentage of death is even lower, accounting for 4% and 0,4%, respectively [7]; in a prospective observational study in a NU setting in South India, approximately 2% of medication errors were life-threatening, although no deaths were reported in that sample [8].

Errors can occur at each stage of the therapeutic course, from drug prescribing to administering [1]. Furthermore, in the neonatal setting, many medications are used off-label and without standardized dosing protocols or specific formulations, which represent other crucial risk factors [9]. To mitigate these problems, various interventions have been proposed and implemented, including the presence of clinical pharmacists into the care team, the use of computerized physician order entry systems (CPOE), and the centralization of high-risk drug preparation in hospital pharmacies. Simpson et al.[10] highlight the utility of adopting an integrated approach among professional figures, such as neonatologists, nurses, and pharmacists. More recently, Ghezaywi et al.[11] suggest combining both human and technology-based strategies to significantly reduce MEs. The awareness of being involved in an improvement process can have a beneficial impact on reducing errors, a phenomenon known as the Hawthorne effect [12].

### General aspects

In our study, geographical location emerged as a strong determinant of error reporting. NUs located in the northern regions of Italy have markedly higher odds of reporting ≥ 5 MEs/year compared to those in the South and Islands. This pattern may reflect regional differences in error detection practices, reporting culture, or system complexity.

No correlations were found between error rates and the number of beds in NUs. This suggests that other factors, such as organizational culture, staffing, or reporting protocols, may play a more critical role in the incidence or declaration of errors. Nonetheless, Bond et al. [13] have found a linear correlation between hospital bed capacity and adverse events or MEs.

### Technological tools

Medication Administration Records (MARs) are essential tools for the daily therapeutic process. Our data show that more than two-thirds of NUs still use paper-based MARs, which poses a significant risk of illegibility, misinterpretation, or transcription errors [3].

Undoubtedly, digitalization can significantly improve clinical workflow by replacing paper-based MARs with electronic-based documents that are accessible and editable in real-time. This technological shift is expected to substantially reduce the percentage of medication errors [4, 8].

The prescribing process in NUs is a critical phase, where accuracy, up-to-date knowledge, and adequate decision support are essential for ensuring neonatal safety [14]. The majority of Italian NUs use the *2020 App Neofarm-SIN *[4] as a formulary, which our Pharmacotherapy Study Group provides at no cost to all members of SIN. A notable decrease in the utilization of the App for drug prescriptions (61%) was observed in units that reported more than five errors annually, in contrast to units that reported fewer errors (79%). It is concerning that 19% of NUs, mainly in Group 1, still use the 2009 Manual of neonatal pharmacotherapy [5]. This constitutes a knowledge problem, considering dosages that have been revised, new drugs introduced, and outdated ones removed, but more importantly, it may pose a potential safety risk for neonates.

Another emerging problem is that dose calculation is still performed manually or with a calculator in most centers, with less than one-third of centers using specific software. Among these, a tiny minority relies on specialized tools such as DIGISTAT or Excel-based spreadsheets.

### Double-checking during drug prescription and preparation

Double-checking is resource-intensive and time-consuming for doctors and nurses. Studies are needed to better understand “if, and when” double-checking is effective. Nevertheless, the Institute for Safe Medication Practices recommends “the selective and proper use of independent double-checks’, to reduce the risk of ritualizing this process” [15]. In our survey, preparation double-check limited only to HA drugs was more effective in MEs detection than extensive control of all drugs.

Among Italian NUs, we have found that medical doctors mostly double-checked only HA drugs (globally and in each error group). This could be a planned choice to save time during the busy daily job. It is also significant to note that, among centers that perform a double check on all prescriptions, Group 2 showed a lower percentage of double check than Group 1 (15% vs. 25%).

Similarly, about preparation, we found that nurses’ double check in Group 2 is also predominantly focused on HA drugs (59%), conversely, in Group 1, nurses check mainly all drugs (46%). These data seem to support a more thorough review of drug prescriptions and preparation.

Specific drug preparation and administration protocols provide a way to reduce the risk of errors, [9]. Most analysed NUs use internal protocols (84%); specifically, some centers are more focused on a specific group of drugs that, due to their characteristics, are prone to error: HA drugs (43%), emergency drugs (18%), and LASA (4.5%). Drug preparation is performed by nurses on duty in almost all Italian NUs; only 3/89 centers report having dedicated nurses. One NICU delegates it to the Hospital Pharmacy, and one center utilizes a robotic system.

### Parenteral nutrition

Regarding PN preparation, this is typically handled by the pharmacy (52%), with an advantage in terms of security, or by nurses in more than 30% of NUs: specifically, nurses on duty (30%) – who experience an increase in their workload – or dedicated nurses (4.5%). Only a tenth of centers use PN-ready-made standard bags; this is a practical way to start PN with a safe product around the clock. It is reported that 8% of NUs modify those PN bags in their wards, despite some suspicions about the security of metabolic and microbiological outcomes when modified.

The study by Hermanspann et al. [16] underscores the necessity of CPOE and clinical guidelines to reduce medication errors, even for PN. They also emphasize the importance of a revision by a clinical pharmacist. Many Italian NUs are using a CPOE system for PN [17].

### Patient identification

A peculiar aspect to take into consideration is that in a great majority of NUs, the same person prepares and administers the drugs. This aspect deserves some reflection. Although this action is necessary for legal reasons, conversely, it could easily lead to error (using the wrong syringe or administering to the wrong patient), mainly in an emergency setting [18], if a proper patient identification strategy is not employed. It was found that only one-third of surveyed NUs use a patient identification strategy.

The use of the *‘‘5 rights’’ items protocol* (right patient, right drug, right dose, right time, and right administration route) supported by double-checking procedures, standardized checklists, and staff education, as described by Marafu et al., can trigger a reduction of ME[19].

## Error reporting

In 80% of Italian centers, errors are voluntarily noted. The presence of digital systems for therapeutic error reporting was also positively associated with high error reporting rates. Similarly, units with anonymous error reporting noticed ME more easily and were more frequently allocated in the high-error reporting category. These associations reasonably reflect more robust surveillance, and lower thresholds for MEs reporting in technologically advanced or culturally high-level environments. Prescription is the most prevalent error, both globally and in the 2 groups of error reporting. This finding is consistent with data reported in the literature [3, 20, 21]. Specifically, in centers with ≥5e/y, prescription errors occurred with the same frequency as preparation, highlighting that MEs are concerns that equally involve neonatologists and nurses [22]. Surprisingly, we have not found any significative correlation between the shift type (day vs. night or weekdays vs. holidays) and the error rate [23].

## Communication aspects

Communication with parents is an integral part of care because parents require transparent and honest information [24].Open disclosure of errors is now widely regarded as a moral and professional duty; in many countries, it has become a formal obligation supported by legal guidelines and standards. Laws such as *Duty to Disclosure Statutes* have been enacted in some jurisdictions to protect physicians and encourage transparent communication [25]. While admitting an error may initially strain the physician-parent relationship, it often deepens trust and reinforces the therapeutic alliance in the long term. A recent French survey [26] has reported a disclosure rate of harmful errors at 59%, which is slightly lower than the 70% rate found in a 2020 Finnish analysis [27]. Only 21% of our respondents always communicate therapeutic errors to the families. In most NUs, there is considerable discretion in declaring mistakes, depending on whether the error poses a problem for the patient. A large portion prefers not to inform parents, for various reasons: “no consequence for the infant”, to avoid work-related problems or for fear of legal issues” [26, 28]. The national high rate of lawsuits against nurses and doctors is too often specious, undermining the transparency of the relationship with parents, as evidenced above by the low percentage of error disclosure.

### Proactive attitude

The MEs reporting system plays a crucial role in understanding the magnitude and specific aspects of this problem. Then, an effective medical error analysis is a necessary step for optimizing the NU’s medical drug order system strategy, outlining a virtuous circle of analysis and readjustment [7, 8, 29]. Unfortunately, we have found that more than 80% of NUs do not have an annual error report, and 55% do not provide for reevaluation of corrective measures.

Our study includes some limitations. The survey focused on therapeutic errors; however, the numbers are estimated values due to the lack of an annual internal report among Italian NUs. Furthermore, we investigated MEs that occurred in 2020 through the survey that was administered between 2022 and 2023. These factors may have introduced subjective emotional and memory biases.

## Conclusion

It is necessary to recognize that MEs are travel companions during clinical practice in NUs, even if though “zero errors” remains the desired goal. There is still a lack of standardized definitions of ME in the literature. This aspect hampers a consistent univocal measurement of errors and the clear evaluation of effective interventions. The most frequent types of errors declared involve the drug prescribing and preparing phases, emphasizing the need for improved accuracy and mutual control between doctors and nurses. Syringe labelling and patient identification systems are other steps to enhance the NUs’ security procedures. The choice of interventions for improving medication safety should be tailored to local resources, type and severity of errors that occur within every NU. The regional discrepancy we have detected may reflect methodological differences in error reporting, access to technological resources, or organizational practices. To support a culture of healthcare system safety, collecting data from real practice, it could be worthwhile to create a standardized, simple, and anonymous digital reporting tool embedded directly into the 2020 Neofarm-SIN app. This “unified error-reporting form” could facilitate faster notifications across all regions, improving surveillance, integrating official modules often underused because too complex. With this survey, the Italian Pharmacotherapy study group would promote a culture of clinical risk management, shifting the paradigm from a sense of guilt to a critical analysis of facts, with the aim of improving the working environment and reducing preventable medical errors. Oscar Wilde stated, “Experience is the name everyone gives to their mistakes” [30]. We believe that we can learn from our errors, and even more from re-evaluating the corrective actions taken. This is the key to achieving better standards of care for our patients.

## Supplementary Information

Below is the link to the electronic supplementary material.

Supplementary Material 1

Supplementary Material 2

## Data Availability

The datasets used and analyzed during the current study data are available from the Neonatal Pharmacotherapy Study Group of the Italian Society of Neonatology (SIN) on reasonable request and with permission of the Italian Society of Neonatology board.

## Declarations

### Pharmacotherapy Study Group of the Italian Society of Neonatology (SIN)

Elena Sorrentino^9^, Genny Raffaeli^10,11^, Sabrina Palamides^12^, Rocco Agostino^13^

^9^Neonatology and NICU, Ospedale S. Pietro Fatebenefratelli, Rome, Italy

^10^NICU, Fondazione IRCCS Ca’ Granda Ospedale Maggiore Policlinico, Milan, Italy

^11^Department of Clinical Sciences and Community Health, Università degli Studi di Milano, Milan, Italy

^12^Neonatology and NICU, Ospedale S. Giovanni Addolorata, Rome, Italy

^13^Ethics Committee for drugs, IRCCS Ospedale Pediatrico Bambino Gesù, Rome, Italy
