# Supplementary material for: Medical error in neonatal units: an Italian survey on national frequency and types of error
Source: Ital J Pediatr. 2026 Aug 11;52:161. doi: 10.1186/s13052-026-02306-6 (PMC13746486; doi:10.1186/s13052-026-02306-6)
Supplement: Supplementary file 1 [file 13052_2026_2306_MOESM1_ESM.docx]

**SURVEY ON THERAPEUTICAL AND CLINICAL ERRORS**

**1. Hospital of affiliation**……………………………………………………………….………….………….... 2.**City** ……………………………………………………….

**3. Email of the person completing the form**……………………………………………………

**4. What is the number of beds in your Unit:**

- Neonatal Pathology: ………………..…………..
- NICU + Intermediate Care: ……………………
- Nursery: ……………………………………………….

**5. In your Unit, is a single therapy sheet used (intended as the sole document where the physician prescribes/updates the therapy and the nurse records the administration)?**

- Yes, paper-based
- Yes, electronic
- No, not used"

**6. In your Unit, when prescribing a drug, the physician usually refers to the following sources to determine the dosage** (multiple answers allowed)**:**

- The guidelines on drug use of the Unit of affiliation
- The printed version of the 2009 Neonatal Pharmacotherapy Manual of the Italian Society of Neonatology (SIN)
- The SIN Neonatal Pharmacotherapy mobile App: *NeoFarm*
- Other printed neonatal pharmacotherapy formularies
- Other electronic/online neonatal pharmacotherapy formularies

**7. In your Unit, is there an automatic calculation system to support prescribing?**

- Yes, calculations are performed using dedicated software
- Yes, calculations are performed using another system (please specify) ……………………………………………………
- No, dosage calculations are performed manually or with a calculator

**8. In your Unit, is a double-check of the prescription required?**

- No
- Yes, for all drugs, by another physician
- Only for High-Alert medications (HA), by another physician
- Yes, for all drugs, by a pharmacist
- Only for High-Alert medications, by a pharmacist

**9. In your Unit, is a double-check of drug preparation required?**

- No
- Yes, for all drugs, by another nurse
- Only for High-Alert medications (HA), by another nurse
- Yes, for all drugs, by a pharmacist
- Only for High-Alert medications, by a pharmacist

**10. In your Unit, are there specific written procedures for the preparation and administration of particular therapies** (multiple answers allowed)**?**

- No
- Yes, for High-Alert medications (HA)
- Yes, for LASA (look-alike/sound-alike) medications
- Yes, for prescribing medications in emergencies
- Yes, also in other cases (please specify): ………………………………………………………….

**11. In your Unit, are there written guidelines for drug dilution?**

- No, there are no Unit guidelines for drug dilution
- Yes, there are Unit guidelines for the dilution of all frequently used drugs
- There are guidelines for the dilution of all frequently used drugs, specifically for ELBW or VLBW neonates
- In the absence of Unit guidelines, the dilutions usually follow those proposed in neonatal formularies (paper or electronic, e.g., ward software or the *NeoFarm* App)

**12. If the dilution recommended by the guidelines/formularies is not suitable for the patient, which professional can modify it?**

- It is modified independently by the nurse
- It is modified independently by the physician
- It is modified collaboratively by the physician and the nurse

**13. In your Unit, who is responsible for preparing medications (not in emergencies) for hospitalized patients?**

- The Unit’s nurses
- One or more nurses from the Unit dedicated exclusively to medication preparation
- The Hospital Pharmacy service
- A pharmacist dedicated to the Unit, working within the Unit’s facilities
- A robotic medication preparation system is in place, either in the Pharmacy or the Unit

**14. In your Unit, who is responsible for preparing Parenteral Nutrition for hospitalized patients?**

- The Unit’s nurses
- One or more nurses from the Unit dedicated exclusively to Parenteral Nutrition preparation
- The Hospital Pharmacy service
- A pharmacist
- A physician from the Unit
- Ready-to-use standard bags, modified in the Unit, are used
- Ready-to-use standard bags, modified in the pharmacy, are used
- Parenteral Nutrition is not available / not administered in our Unit

**15. In your Unit, is the person who prepares the medication usually the same as the one who administers it?**

- No
- Yes

**16. Is there a drug/patient labeling system in your Unit?**

- Yes, a drug/patient identification system is in place (prescription/bracelet)
- Yes, an automatic bedside identification system is in place (e.g., unique barcode scanning) to support the therapeutic process (single-dose–patient identification)
- No, because the drug is prepared and administered immediately
- No, for another reason (please specify) …………………………………………………………………

**17. Is there a system for detecting medication errors in your Unit?**

- Yes, based exclusively on voluntary error reporting/incident reports
- Yes, also involving chart reviews or other systems
- No

**18. If available, how can staff report a Medication Error (ME) or submit an incident report?**

- On a paper form
- On an electronic platform
- By e-mail
- With guaranteed anonymity
- To the Unit Director, Head Nurse, or Nursing Coordinator
- To the Risk Manager or facilitators of the clinical risk management system

**19. A medication error is a preventable event that may cause or lead to inappropriate use of a drug or patient harm. Based on this definition, how many medication errors were detected, or are estimated to have occurred (if no detection system is in place), in your Unit in 2020?**

- <5
- 5–10
- 10–20
- >20

**20. Which type of error was most prevalent (or estimated to be most prevalent) in your Unit in 2020?**

- Prescription error
- Transcription error
- Preparation error
- Identification error
- Administration error

**21. In your Unit, during which shift did medication errors occur most frequently?**

- Day shift
- Night shift
- Weekday shift
- Holiday shift
- There were no significant differences in the incidence of medication errors between shifts
- I don’t know

**22. What do you consider to be a realistic estimate of the frequency of reported medication errors or those for which an incident report was filed?**

- 0–25%
- 25–50%
- 50–75%
- 75–100%

**23. What do you think is the most frequent reason for not reporting a medication error?**

- Fear of consequences in the workplace
- Feeling that reporting is unnecessary if no clinical harm occurred to the neonate
- Fear of legal repercussions
- Desire to avoid additional workload that reporting might entail

**24. In the event of a medication error, are the parents informed?**

- Never or almost never
- Sometimes
- Always
- Only in cases where the error has consequences for the neonate (including the need to modify care or therapy)
- It depends on the type of error (consequences)

**25. In the event of a medication error, which professional most frequently informs the parents of the incident?**

- The Unit Director
- The attending or on-call physician
- The Nursing Coordinator / Head Nurse
- Another professional (please specify

**26. Is an annual report on medication errors prepared in your Unit and presented/discussed with the team and the Hospital Management?**

- No
- Yes

**27. Is there a monitoring system in your Unit to verify the effectiveness of corrective actions implemented following the analysis of medication errors?**

- No
- Yes

**28. How many training courses (including webinars) do you attend during the calendar year?**

- None
- Only mandatory courses organized by the facility
- <5
- 5-10

**29. You choose training courses based on:**

- Your sub-specialization
- Issues encountered in your daily work (please specify the type of issue)
- Directives from your supervisor
- Other (please specify)

**30. Are neonatal resuscitation courses and/or simulations organized in your hospital?**

- Yes
- No

**31. If yes, how often are they held?**

- Semiannual
- Annual
- Biennial
- Other (please specify)

**32. Prenatal counseling is carried out:**

- Systematically, for every high-risk birth, jointly by the obstetrician and neonatologist, with completion of the Prenatal Card (pre-filled form)
- Only for preterm births
- Rarely
- Only if requested by the neonatologists

**33. In your hospital, the observation of healthy newborns immediately after birth is carried out:**

- In the delivery room, by the midwife on duty, during bonding with the mother
- In the rooming-in area, by the midwife/nurse on duty
- In the nursery, by the midwife/nurse on duty

**34. Is there an observational chart for the newborn after birth and in the following hours?**

- Yes
- No

**35. If it exists, are the newborn's parameters recorded and signed?**

- Yes
- No

**36. If yes, how often and by whom?**

- Every 15–30 minutes by the midwife
- Every 15–30 minutes by the nurse
- Every 15–30 minutes by the neonatologist

**37. In your hospital, when is the first examination of a 'healthy' newborn performed?**

- In the delivery room, in the neonatal area
- 30 minutes after birth
- 2 hours after birth
- As soon as the newborn arrives in the nursery

**38. Are procedures carried out to prevent 'Sudden Unexpected Postnatal Collapse' (SUPC)?**

- Yes
- No, I don’t know what it is
- Only in specific situations

**39. Are clinical audits carried out following 'near miss events' or medication errors?**

- Always
- Never
- Rarely

**40. Which method do you consider most appropriate for acquiring non-technical skills (e.g., communication, teamwork, problem-solving, etc.)?**

- Interactive seminar
- Lectures
- Case studies
- Other (please specify)
- Small group work
- Simulation

**41. Which non-technical skills would you like to develop? (multiple answers allowed)**

- Patient communication
- Leadership improvement
- Teamwork
- Problem-solving
- Other (please specify)

**42. Is scheduled maintenance and safety check of medical equipment in your Unit carried out:**

- By the specialist upon specific request
- Periodically by the specialist according to the contract
- By clinical engineering upon specific request
- Periodically by clinical engineering

**43. Is there a patient evacuation and mobilization plan in your Unit?**

- No
- Yes, but it has never been standardized
- Yes, a written procedure exists

**44. Any comments on the questionnaire………………………………………………………………………………………………………………………………………………………………………………….**
