# Supplementary material for: Medical error in neonatal units: an Italian survey on national frequency and types of error
Source: Ital J Pediatr. 2026 Aug 11;52:161. doi: 10.1186/s13052-026-02306-6 (PMC13746486; doi:10.1186/s13052-026-02306-6)
Supplement: Supplementary file 2 [file 13052_2026_2306_MOESM2_ESM.docx]

|  | Drug |
| --- | --- |
| 1 | Enoxaparin |
| 2 | Sodium Heparin (unfractionated) |
| 3 | Human Insulin (regular insulin) |
| 4 | ConcentratedPotassiumChloride |
| 5 | Rocuronium |
| 6 | Norepinephrine |
| 7 | Fentanyl |
| 8 | Morphinesulfate |
| 9 | Methadone |
| 10 | Midazolam |

**High Alert**

**LASA**

|  | Drug A | Drug B |
| --- | --- | --- |
| 1 | AMPLITAL 1 g vial | AMPLITAL 500 mg vial |
| 2 | ATROPINE SULFATE 0.5 mg vial MONICO | ADRENALINE 1 mg vial MONICO |
| 3 | BROMAZEPAM RATIO 20 ml drops | DELORAZEPAM RATIO 20 ml drops |
| 4 | BROMAZEPAM RATIO 20 ml drops | DIAZEPAM RATIO 20 ml drops |
| 5 | CALCIUM CHLORIDE 1 g 10 ml vial MONICO | CALCIUM GLUCONATE 1000 mg vial MONICO |
| 6 | CALCIUM GLUCONATE 1 g 10 ml vial BIOINDUSTRIA | CALCIUM CHLORIDE 1 g 10 ml vial BIOINDUSTRIA |
| 7 | CALCIUM CHLORIDE 1 g 10 mLvial | CALCIUM GLUCONATE 1000 mg vial |
| 8 | CALCIUM CHLORIDE vial | SODIUM CHLORIDE 0.9% vial |
| 9 | FENTANYL HEXAL 25 µg/h patch | FENTANYL HEXAL 100 µg/h patch |
| 10 | FLUCONAZOLE KABI 100 mLvial | SODIUM CHLORIDE KABI 0.9% 100 mLvial |

**Emergency Drugs**

|  | Drug |
| --- | --- |
| 1 | Adrenaline (epinephrine) |
| 2 | Amiodarone |
| 3 | Adenosine |
| 4 | Atropine |
| 5 | Naloxone |
| 6 | Midazolam |
| 7 | Diazepam |
| 8 | Lidocaine |
| 9 | Furosemide |
| 10 | Noradrenaline (norepinephrine) |

*References:*

*Italian Ministry of Health. (2010, August).* Recommendation No. 12: Prevention of medication errors involving “Look-Alike/Sound-Alike” (LASA) drugs.

*Italian Ministry of Health. (2008, March).* Recommendation No. 7: Prevention of death, coma, or serious harm resulting from medication errors.

*Italian Medicines Agency (AIFA). (2023).* High-alert medications: Identification and management of clinical risk.

*Italian Medicines Agency (AIFA). (2024).* Good practices for the management and administration of medications in emergency settings.
